# Alleviation of Anxiety/Depressive-Like Behaviors and Improvement of Cognitive Functions by *Lactobacillus plantarum* WLPL04 in Chronically Stressed Mice

**DOI:** 10.1155/2021/6613903

**Published:** 2021-01-30

**Authors:** Xuan Sun, Hong-Fei Zhang, Chao-Lin Ma, Hua Wei, Bao-Ming Li, Jie Luo

**Affiliations:** ^1^School of Life Sciences, Nanchang University, Nanchang 330031, China; ^2^Institute of Life Science, Nanchang University, Nanchang 330031, China; ^3^State Key Laboratory of Food Science and Technology, Nanchang University, Nanchang 330029, China; ^4^School of Public Health and Key Laboratory of Preventive Medicine, Nanchang University, Nanchang 330006, China

## Abstract

**Background:**

Intestinal microorganisms play an important role in regulating the neurodevelopment and the brain functions of the host through the gut-brain axis. *Lactobacillus*, one of the most representative intestinal probiotics, produces important effects on human physiological functions. Our previous studies reveal that the *Lactobacillus plantarum* WLPL04 has a series of beneficial actions, such as antiadhesion of pathogens, protection from the harmful effect of sodium dodecyl sulfate, and anti-inflammatory stress on Caco2 cells. However, its effects on brain functions remain unknown. The present study aims to evaluate the potential effect of *L. plantarum* WLPL04 on anxiety/depressive-like behaviors in chronically restrained mice.

**Methods:**

Newly weaned mice were exposed to chronic restraint stress for four weeks and raised daily with or without *L*. *plantarum* WLPL04 water supplement. Animals were behaviorally assessed for anxiety/depression and cognitive functions. The 16S rRNA sequencing was performed to analyze the intestinal microbiota structure. The levels of the medial prefrontal cortical (mPFC) brain-derived neurotrophic factor (BDNF)/tropomyosin-related kinase B (TrkB) and serum 5-hydroxytryptamine (5-HT) were examined using Western blot and enzyme-linked immunosorbent assay.

**Results:**

The chronic stress-induced anxiety/depressive-like behaviors and cognitive deficits were significantly alleviated by the *L*. *plantarum* WLPL04 treatment. The 16S rRNA sequencing analysis showed that the chronic stress reduced the diversity and the richness of intestinal microbiota, which were rescued by the *L*. *plantarum* WLPL04 treatment. The levels of BDNF and TrkB in the mPFC and the concentration of 5-HT in the serum remained unchanged in chronically restrained mice treated with the *L. plantarum* WLPL04.

**Conclusions:**

The *L. plantarum* WLPL04 can rescue anxiety/depressive-like behaviors and cognitive dysfunctions, reverse the abnormal change in intestinal microbiota, and alleviate the reduced levels of 5-HT, BDNF, and TrkB induced by chronic stress in mice, providing an experimental basis for the therapeutic application of *L. plantarum* on anxiety/depression.

## 1. Introduction

The chronic stress in early life increases the susceptibility to a range of psychopathologies, including depression and anxiety [1]. Traditional psychotropic medications are controversial partly because the long-term effects to developing nervous systems have not been clearly established [2]. Recently, accumulating evidence indicates the existence of a microbiome-gut-brain axis and strong bidirectional communications among these structures [3, 4]. Stress in early life can alter the enteric microbiota [5], and intestinal bacterial infection can induce anxiety-like behaviors and cause memory deficits [6, 7]. Thus, the regulation of the intestinal microbiota is suggested to be an interesting strategy for the development of new therapy for mental diseases [8].

Probiotics are a group of active microorganisms and confer health benefits to the host via active interactions with endogenous microbiota and gut cells when provided in appropriate amount [9]. Probiotics are also beneficial to patients suffering from psychiatric illness. In 2005, Logan and Katzman have used probiotics as adjunct therapy for depression for the first time [10]. Some strains of *Lactobacillus* and *Bifidobacterium* are shown to alleviate mood disorders and prevent stress-induced alterations in colonic microbiota [8, 11].

Lactic acid bacteria are regarded as safe and beneficial probiotics that may help prevent constipation, irritable bowel syndrome or Crohn's disease, and asthma or eczema in children [12, 13]. A recent study shows that the *Lactobacillus plantarum* NDC75017 alleviates the learning and the memory deficit in aging rats by reducing the mitochondrial dysfunction [14]. The *Lactobacillus plantarum* MTCC1325 strain, which produces acetylcholine, has potential antioxidant and anti-Alzheimer activities against the D-galactose-induced Alzheimer's disease [15, 16]. Most *L. plantarum* strains are isolated from fermented food, whereas the strains isolated from human breastmilk have some special features, such as regulating natural and acquired immune responses, treating infectious mastitis, having antimicrobial properties, and having beneficial effects for infants [17–19]. A previous study shows that the *L*. *plantarum* WLPL04 isolated from human breastmilk can increase the capabilities of human body against pathogens and may be a candidate probiotic for promoting host health [20]. The present study aims to assess the effects of *L*. *plantarum* WLPL04 on anxiety/depressive-like behaviors and cognitive functional deficits induced by chronic stress in young adult mice and analyze the underlying mechanism.

## 2. Materials and Methods

### 2.1. Animals

A total of 30 male C57BL/6 mice (approximately four weeks old at the start of the experiments) were used. All mice were randomly assigned to three groups (10 mice per group), including one control group and two chronic restraint-stressed groups. One of the stressed groups was provided with normal drinking water, and the other stressed group was provided with normal drinking water and *L*. *plantarum* WLPL04, which was kindly provided by State Key Laboratory of Food Science and Technology, Nanchang University, China. The final concentration of the *L*. *plantarum* WLPL04 in drinking water was 10^9^ CFU/mL. The mice were housed in cages (3 or 4 mice per cage) under a constant temperature (23°C–25°C) and a 12 h light/dark cycle with ad libitum access to food (SHOOBREE, SPF-grade chow, Jiangsu Xietong Pharmaceutical Bio-Engineering Co., Nanjing, Jiangsu, China) and water (sterilized drinking water). Mice were placed in a plastic restrainer in their home cages for 3 h daily (from 11:00 to 14:00) for 28 consecutive days to establish chronic restraint stress [21]. All experiments were carried out in accordance with the principles of laboratory animal care and use approved by the Nanchang University Animal Care and Use Committee Guidelines.

### 2.2. Behavioral Assessments

Prior to behavioral tests, all mice were handled for five days and received 1 h accommodation to the experiment room before testing. The open field, elevated plus maze, and forced swimming tests were performed to analyze anxiety/depressive-like behaviors, and the novel object recognition and Barnes maze tests were used to evaluate the cognitive functions of the medial prefrontal cortex (mPFC).

#### 2.2.1. Open Field Test

The open field test was performed in accordance with previously described procedures [22]. Each mouse was placed in an open field arena (45 cm × 45 cm × 40 cm, Med Associates, Vermont, USA) and allowed to freely explore the arena for 10 min to carry out the test. The locomotion of the mouse was recorded using a video capture software. The total distance and the routine traveled in the arena were measured. The open field arena was cleaned with 75% ethanol after each use.

#### 2.2.2. Elevated Plus Maze Test

The elevated plus maze test was performed in a gray plastic cross-shaped maze (Med Associates, Vermont, USA) with 1.0 m elevation from the floor, two open arms (35 cm × 7 cm), and two closed arms (35 cm × 7 cm × 40 cm). The arms were connected by the center platform (7 cm × 7 cm). A mouse was placed in the intersection of the open and closed arms, facing an open arm, and allowed to freely explore the maze for 10 min. The behavioral exploration and the time spent in the open and the closed arms were video recorded for analysis. The maze arms were cleaned with 75% ethanol after each use.

#### 2.2.3. Forced Swimming Test

The forced swimming test was performed in a Plexiglas cylinder (25 cm in height and 10 cm in diameter) containing water at height of 10 cm and temperature of 22°C–25°C. The water was changed between trials. Each mouse was allowed to swim for a maximum of 6 min, and the immobility time was recorded during the last 5 min of the trial. After the trial ended, the mouse was carefully dried with a cloth towel and kept under a heating fan for 1 h before placing back into the home cage.

#### 2.2.4. Barnes Maze Test

**Figure 1:**
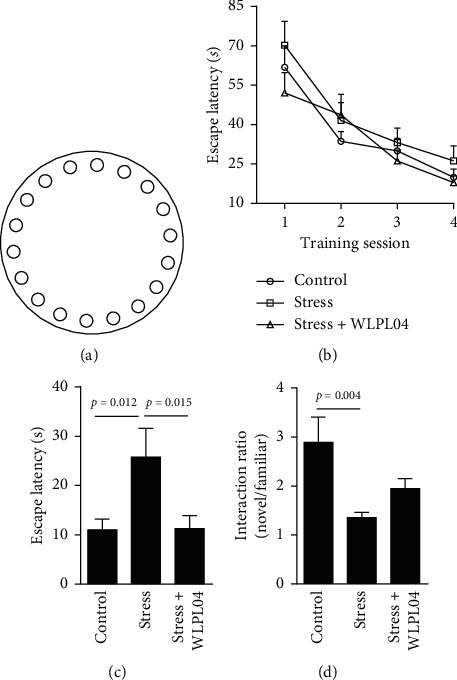
*L*. *plantarum* WLPL04 treatment alleviates the cognitive dysfunctions in chronically stressed mice. (a) Diagram of Barnes maze. (b), (c) Chronic stress impairs spatial memory but not spatial learning in the Barnes maze, and such memory deficit is rescued by the *L*. *plantarum* WLPL04 treatment. (d) Chronic stress impairs the novel/familiar object recognition, and such deficit is reversed by the *L*. *plantarum* WLPL04 treatment. *n* = 7 mice per group.

The Barnes maze task is a spatial memory task. The maze apparatus (Techman, BMT-100, Chengdu, China) was a round platform (75 cm in diameter) with 18 evenly arranged holes (7 cm in diameter, Figure 1(a)). During the training, a target box was placed below one of the holes, which was labeled as the target hole. Training and testing were performed in a 500 lux light environment. The platform was cleaned with 75% ethanol after each use to avoid smelling interference with the next animal.

The day before the training, a mouse was placed in the target hole for 1 min to get familiar with the environment. For the spatial training, target holes were maintained in the same location relative to the extramaze cues on each trial. The trial was started by placing a mouse in the center of the platform. The mouse was covered with a cylinder. After 10 s, the mouse was allowed to freely explore the platform for 3 min. If the mouse found the target hole, the mouse was allowed to stay inside the target box for 30 s. If the mouse failed to find the target hole, the mouse was guided to the target box, and the environment light was turned off simultaneously. The mouse was permitted to stay in the box for 30 s. Each mouse was trained for three 3 trials each day for four consecutive days.

For the spatial memory testing, the target box was removed and each mouse underwent a probe trial on the fifth day. The mouse was placed on the center of the platform and allowed to freely explore the platform for 90 s. The latency to find the target box was recorded.

#### 2.2.5. Novel Object Recognition Test

The novel object recognition task was performed in a square arena (30 cm × 30 cm × 45 cm). Each mouse was placed in the arena to explore for 1 min to get familiar with the environment. One day later, two identical objects were placed in two distinct corners of the arena, and the mouse was allowed to explore the arena for 10 min. On the next day, one of the two identical objects (familiar objects) was replaced by a novel object (nonfamiliar object). The novel object was different in shape and color with the familiar ones. The mouse was placed in the arena to freely explore for 10 min. The exploration behavior of the mouse was video recorded for analysis. The arena was cleaned with 75% ethanol after each use.

### 2.3. DNA Extraction and High-Throughput DNA Sequencing

After the chronic restraint stress, the mouse feces was collected from every cage and immediately placed in 1.5 mL screw-capped tubes for DNA extraction. Prior to the DNA extraction, each sample tube was added with sterilized phosphate-buffered saline (PBS) solution (6 ml, 0.05 M, pH 7.4), shaken for 5–10 min, and centrifuged for 5 min at 500 rpm. The deposits were collected in Eppendorf tubes, and such processes were repeated thrice. The deposits were suspended in 1.0 mL ddH_2_O and centrifuged for 5 min at 14000 rpm. The resulting deposits were dissolved in 200 *μ*l absolute ethanol (precooled at −20°C) and centrifuged for 2 min at 14,000 rpm. The supernatant was discarded, and the process was repeated thrice. The total DNA was extracted from the feces by using the TIANamp Bacteria DNA Kit (TIANGEN, DP302, Beijing, China) in accordance with the manufacturer's instructions. The extracted genomic DNA was sent to Personal, Inc. (Personal Bio Inc., Shanghai, China) for high-throughput sequencing and analysis.

The V3/4 region of the 16S rRNA gene was amplified using universal primers 338F (5′-ACTCCTACGGGAGGCAGCAG-3′) and 806R (5′-GGACTACHVGGGTWTCTAAT-3′). The PCR product was extracted from 2% agarose gels, purified, and quantified. The sequencing was carried out using the Illumina MiSeq platform and 2 × 300 bp reagent kit for paired-end sequencing (GenBank accession number PRJNA673977). Operational taxonomic units (OTUs) were clustered with 97% similarity cutoff, and chimeric sequences were identified and removed using the QIIME analysis tools (Quantitative Insights into Microbial Ecology, v1.8.0; http://qiime.org/).

### 2.4. Enzyme-Linked Immunosorbent Assay (ELISA)

The truck blood was collected and centrifuged at 3400 rpm for 20 min to measure the 5-HT level. The supernatant was collected and stored at −80°C for further analysis. The 5-HT level was measured using the commercial ELISA kit (ab133053, Abcam, Cambridge, UK). In brief, varying concentrations of standard and sample solutions were added into the ELISA plates. All ELISA measurements were performed in two replicates.

### 2.5. Protein Extraction and Western Blot

Brain tissues were quickly removed, washed with PBS, and homogenized with phenylmethanesulfonyl fluoride (Sigma-Aldrich, 78830, Wisconsin, USA). The total protein concentration was measured using the BCA protein assay kit (Thermo Scientific, 23235, New York, USA). Protein extracts were used for Western blot to quantify the levels of brain-derived neurotrophic factor (BDNF), tropomyosin-related kinase B (TrkB), and glyceraldehyde-3-phosphate dehydrogenase (GAPDH). After the measurement and the adjustment of protein concentration, samples were added with 4x loading buffer, heated at 100°C for 10 min, and loaded onto the SDS–PAGE. Proteins were transferred onto polyvinylidene difluoride membranes (Merck Millipore, ISEQ00010 and IPVH00010, Massachusetts, USA) for 2 h at 56 V in the transfer buffer. Membranes were blocked with 5% nonfat milk in Tris-buffered saline (TBST) for 2 h at room temperature on an orbital shaker. The membranes were then cut into several parts in accordance with the goal protein molecular weight and incubated with primary antibodies (anti-BDNF antibody, 1 : 1000, Abcam, ab108319, Cambridge, UK; anti-TrkB antibody, 1 : 5000, Abcam, ab187041, Cambridge, UK; and anti-GAPDH antibody, 1 : 1000, BioRad, MCA4739, California, USA) overnight at 4°C. The membranes were washed thrice with TBST (5 min each time) and incubated with horseradish peroxidase- (HRP-) conjugated IgG secondary antibodies (goat anti-mouse IgG HRP, 1 : 3000, Cw0102s, Beijing, China; and goat anti-rabbit IgG HRP, 1 : 3000, Cw0103s, Beijing, China) for 2 h at room temperature. Signals were visualized using the Gel DocTM EZ System (BioRad, California, USA), and the relative levels of BDNF, TrkB, and GAPDH proteins were analyzed using the “Image J” Software (https://imagej.nih.gov/ij/).

### 2.6. Statistical Analysis

Statistical analysis was performed using the GraphPad Prism 6. Continuous variables were presented as the mean ± standard error of the mean. Results were analyzed using unpaired one-way and two-way ANOVA.

The Chao1 index was determined as follows:

(1) $$\begin{array}{c} S_{\text{Chao}1}=S_{\text{obs}}+\frac{n1\left(n1-1\right)}{2\left(n2+1\right)}, \end{array}$$

where *S*_Chao1_ is the estimated number of OTUs (operational taxonomic units), *S*_obs_ is the observed number of OUTs, *n*1 is the number of OTU that have one sequence, and *n*2 is the number of OTU that have two sequences.

The Shannon index was commonly calculated as follows:

(2) $$\begin{array}{c} H_{\text{Shannon}}=-\overset{R}{\underset{i=1}{\sum }}p_{i}ln\;p_{i}, \end{array}$$

where *p*_*i*_ is the proportion of characters belonging to the *i*^th^ type of letter in the string of interest, and *R* denotes the actual number of types.

## 3. Results and Discussion

### 3.1. *L*. *plantarum* WLPL04 Rescues Stress-Induced Anxiety/Depressive-Like Behaviors

**Figure 2:**
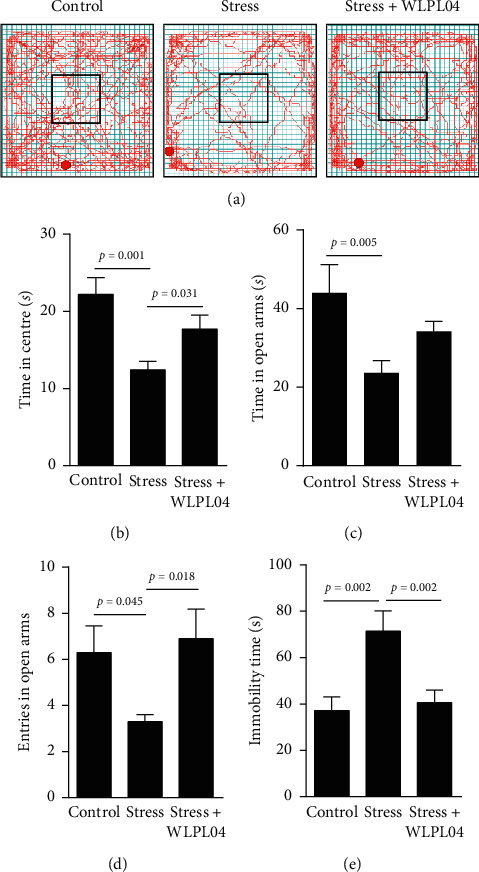
*L*. *plantarum* WLPL04 treatment reduces anxiety-like behaviors in chronically stressed mice. (a) The moving trajectory of the three groups of mice in the open-field test. (b) Chronically stressed mice exhibit significantly (*p* < 0.01) less time in the central area of the open field, and such behavioral phenotype is alleviated by the *L*. *plantarum* WLPL04 treatment. Chronically stressed mice have spent (c) significantly (*p* < 0.01) less time and (d) significantly (*p* < 0.05) fewer entries into the open arms in the elevated plus maze test, and such behavioral phenotypes are alleviated by the *L*. *plantarum* WLPL04 treatment. (e) Chronically stressed mice demonstrate significantly (*p* < 0.01) longer immobility time, and such behavioral phenotype is alleviated by the *L. plantarum* WLPL04 treatment. *n* = 7 – 12 mice per group.

The behavioral assessment with the open-field test showed that the chronically stressed mice spent significantly less time in the central area of the open field compared with the control mice, indicating that the chronically stressed mice exhibited the anxiety phenotype. This phenotype was not observed in the stress + WLPL04 group of mice (Figures 2(a) and 2(b), control: 22.22 ± 2.159 s; stress: 12.41 ± 1.115 s; stress + WLPL04 : 17.73 ± 1.798 s; and one-way ANOVA, *F* (2, 27) = 7.928, *p* = 0.0020).

The elevated plus maze test showed that the *L*. *plantarum* WLPL04 treatment could rescue the chronic stress-induced anxiety phenotype. The stressed mice spent significantly less time (control: 43.93 ± 7.197 s; stress: 23.61 ± 3.078 s; stress + WLPL04 : 34.13 ± 2.552 s; and one-way ANOVA, *F* (2, 27) = 4.571, *p* = 0.0195) and executed significantly less entries (control: 6.300 ± 1.146; stress: 3.300 ± 0.3000; stress + WLPL04 : 6.900 ± 1.286; and one-way ANOVA, *F* (2, 27) = 3.651, *p* = 0.0395) in the open arms of the maze compared with the control mice. This anxiety-like phenotype was not observed in the stress + WLPL04 group of mice (Figures 2(c) and 2(d)).

The forced swimming test was performed to examine the effect of the *L. plantarum* WLPL04 treatment on the depressive-like behavior. The stressed mice had significantly more immobility time (control: 37.17 ± 5.846 s; stress: 71.43 ± 8.680 s; stress + WLPL04 : 40.57 ± 5.467 s; and one-way ANOVA, *F* (2, 32) = 7.670, *p* = 0.0019) compared with the control mice, indicating that the chronically stressed mice exhibited the depressive-like phenotype. This phenotype was not observed in the stress + WLPL04 group of mice (Figure 2(e)).

Overall, these results suggested that the *L. plantarum* WLPL04 treatment could alleviate the anxiety and the depressive-like behavioral phenotypes induced by chronic stress.

### 3.2. *L*. *plantarum* WLPL04 Alleviates Stress-Induced Cognitive Functional Deficits

The Barnes maze and novel object recognition tests were performed to examine whether the *L*. *plantarum* WLPL04 treatment could alleviate the cognitive deficits induced by the chronic restraint stress. In the Barnes maze test, which measured the hippocampus and prefrontal cortex-dependent spatial learning and memory, the stressed and the control mice performed equally well during the training, indicating an intact ability of spatial learning (session 1: control: 61.85 ± 8.202 s; stress: 70.22 ± 9.081 s; stress + WLPL04 : 52.10 ± 7.771 s; session 2: control: 33.64 ± 3.709 s; stress: 41.59 ± 6.798 s; stress + WLPL04 : 43.70 ± 7.901 s; session 3: control: 30.01 ± 4.748 s; stress: 33.14 ± 5.496 s; stress + WLPL04 : 26.16 ± 4.148 s; session 4: control: 19.95 ± 3.223 s; stress: 26.20 ± 5.703 s; stress + WLPL04 : 17.94 ± 2.660 s; and two-way ANOVA, *F* (6, 162) = 0.6035, *p* = 0.7272). However, in the spatial memory in the probe trial, compared with the control mice, the stressed mice spent a significantly longer time (control: 10.96 ± 2.218 s; stress: 25.76 ± 5.814 s; stress + WLPL04 : 11.28 ± 2.641 s; and one-way ANOVA, *F* (2, 39) = 4.693, *p* = 0.0149) to find the target box, indicating impaired spatial memory. Such impairment was not observed in the stress + WLPL04 group of mice, suggesting the protective effect of the *L. plantarum* WLPL04 on the spatial memory consolidation (Figures 1(b) and 1(c)).

In the novel object recognition test, which measures the prefrontal cortex-dependent cognitive function [23, 24], the control mice preferred to interact with the novel objects. By contrast, the stressed mice spent equal time interacting with novel and familiar objects, indicating a deficit in the novel object recognition. Such deficit was not observed in the stress + WLPL04 group of mice, suggesting that the *L*. *plantarum* WLPL04 could protect the cognitive ability for novel object recognition (Figure 1(d), control: 2.889 ± 0.5159; stress: 1.354 ± 0.1111; stress + WLPL04 : 1.946 ± 0.2031; and one-way ANOVA, *F* (2, 18) = 5.620, *p* = 0.0127).

Overall, these results suggested that the *L*. *plantarum* WLPL04 treatment could alleviate the cognitive deficits induced by chronic stress.

### 3.3. *L. plantarum* WLPL04 Reverses the Reduction in the Intestinal Microbiota Diversity Caused by Stress

**Figure 3:**
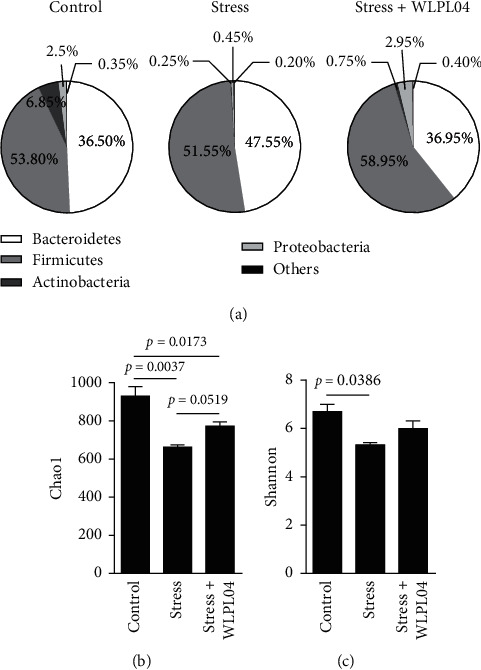
*L*. *plantarum* WLPL04 treatment rescues the negative change in the intestinal microbiota. (a) Aggregate microbiota composition at the phylum level in the fecal samples of experimental mice. Chronic stress significantly reduces the number of Firmicutes, and such negative effect is reversed by the *L*. *plantarum* WLPL04 treatment. Bacterial diversity and species richness as indicated by the (b) Chao1 and the (c) Shannon indices. The chronic stress tends to destroy the diversity and destroy the species richness, and such negative effects are rescued by the *L*. *plantarum* WLPL04 treatment. *n* = 12 mice per group.

The composition of intestinal microbiota was analyzed using the high-throughput DNA sequencing. The analysis of the relative abundance of the detected bacteria within each sample at the phylum level revealed a decrease in the abundance of Firmicutes and Actinobacteria and an increase in the abundance of Bacteroidetes in the stressed mice compared with those in the control mice (Figure 3(a)). Such changes were not observed in the stress + WLPL04 group of mice (Figure 3(a)). The Chao1 and the Shannon analyses showed that the species richness (Chao1) and the microbiota diversity (Shannon) in the feces were reduced by the chronic restraint stress, and such reductions were somehow reversed upon probiotic treatment with the *L*. *plantarum* WLPL04 (Figures 3(b) and 3(c), Chao1: control: 930.0 ± 49.00; stress: 662.0 ± 11.00; stress + WLPL04 : 772.3 ± 21.36; one-way ANOVA, *F* (2, 4) = 18.70, *p* = 0.0093; Shannon: control: 6.700 ± 0.2900; stress: 5.325 ± 0.08500; stress + WLPL04 : 5.990 ± 0.3262; and one-way ANOVA, *F* (2, 4) = 4.606, *p* = 0.0917). Thus, the chronic stress reduced the intestinal microbiota diversity, and such reduction could be reversed by the *L. plantarum* WLPL04 treatment.

### 3.4. *L*. *plantarum* WLPL04 Reverses the Decrease in the Serum 5-HT Level Caused by Stress

**Figure 4:**
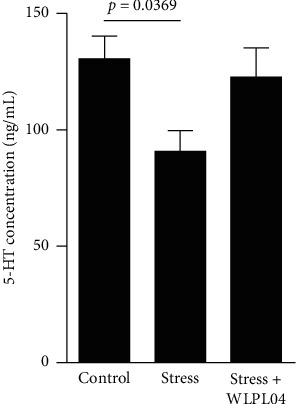
Chronic stress causes a decrease in the serum 5-HT concentration, and such effect is reversed by the *L*. *plantarum* WLPL04 treatment. Three independent experiments were conducted in each sample group.

The 5-HT, a key element in the gut-brain axis, acts as a neurotransmitter in the central and the enteric nervous systems. The 5-HT plays an important role in learning, memory, and emotion regulation in the central nervous systems. The analysis of the serum 5-HT level revealed a significant decrease in the 5-HT level in stressed mice compared with that in the control mice. This decrease was not observed in the stress + WLPL04 group of mice (Figure 4, control: 130.6 ± 9.672 ng/mL; stress: 90.78 ± 8.945 ng/mL; stress + WLPL04 : 122.7 ± 12.65 ng/mL; and one-way ANOVA, *F* (2, 6) = 4.004, *p* = 0.0786), suggesting that the *L*. *plantarum* WLPL04 could produce a protective effect on the chronically stressed mice by maintaining the serum 5-HT at a normal level.

### 3.5. *L*. *plantarum* WLPL04 Prevents the Stress-Induced Decrease in BDNF and its Receptor TrkB

**Figure 5:**
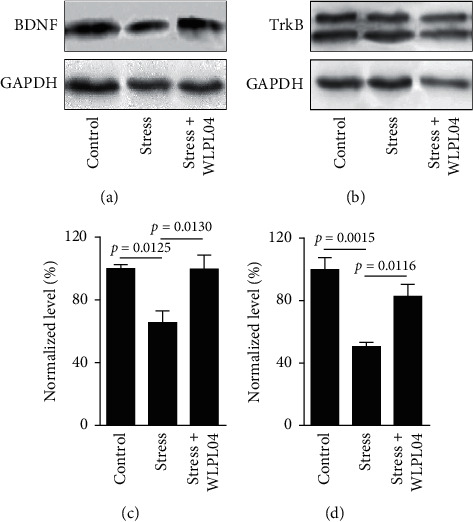
Protein levels of brain-derived neurotrophic factor (BDNF) and tropomyosin receptor kinase *B* (TrkB) in the mPFC of mice. Chronic stress reduces the expressions of (a), (c) BDNF and (b), (d) TrkB, and such effects are rescued by the *L*. *plantarum* WLPL04 treatment. Three independent experiments were conducted in each sample group.

The BDNF, a member of neurotrophic factors, plays an important role in the nervous system development and learning/memory function [25]. Previous reports show that early-life events regulate the expression of neurotrophic factors [26]. Here, the expression levels of BDNF and its receptor TrkB in the mPFC of mice were measured using the Western blot. As shown in Figure 5, the chronic stress significantly reduced the levels of BDNF and the TrKB protein, and these reductions were not detected in the stress + WLPL04 group of mice (BDNF : control: 100.0 ± 2.699%; stress: 65.57 ± 7.401%; stress + WLPL04 : 99.68 ± 9.028%; one-way ANOVA, *F* (2, 6) = 8.182, *p* = 0.0193; TrkB : control: 100.0 ± 7.444%; stress: 50.64 ± 2.565%; sStress + WLPL04 : 82.82 ± 7.694%; and one-way ANOVA, *F* (2, 6) = 15.54, *p* = 0.0042). These results suggested that the *L*. *plantarum* WLPL04 could prevent the stress-induced decrease in BDNF and its receptor TrkB.

## 4. Discussion

The present study shows that the chronic restraint stress induces anxiety/depressive-like behaviors and results in cognitive deficits, causes an abnormal change in the intestinal microbiota, and reduces the levels of 5-HT, BDNF, and TrkB. These changes can be alleviated with the *L*. *plantarum* WLPL04 treatment, providing an experimental basis for the therapeutic application of *L*. *plantarum* on anxiety/depression and cognitive dysfunctions.

A growing body of studies suggests that the probiotic treatment can reverse the stress-induced intestinal dysbiosis and behavioral abnormality. The beneficial effects of probiotics include promoting host digestion, supporting the immune system, and managing the intestinal microbiota [27, 28]. *Lactobacillus* and *Bifidobacteria*, as the representative bacteria of Firmicutes and Actinobacteria, respectively, are reported to reduce anxiety symptoms in patients with chronic fatigue syndrome [29]. A probiotic formulation combining *L*. *acidophilus* Rosell-52 and *Bifidobacterium longum* Rosell-175 exerts a beneficial effect on the gastrointestinal symptoms in individuals affected by chronic stress [30]. The oral administration of *B*. *longum* 1714 or *Bifidobacterium breve* 1205 for six weeks reduces anxiety-like behavioral phenotypes in anxious BALB/*c* mice [31]. Several factors increase the risk of depression, including stress and environment and gut microbiota [32, 33]. Our study shows that four-week chronic restraint stress could cause depression. Previous studies show that gut microbiota can modulate depressive-like behavior. Mice treated with a combination of *Lactobacillus helveticus* R0052 and *Bifidobacterium longum* R0175 present improvements in depression-like behavior [34]. CCFM1025 treatment significantly reduced anxiety and depression-like behaviors induced by chronic unpredictable mild stress [35]. Mice received 1 × 10^9^ cfu·*L. rhamnosus* daily for four weeks could alleviate anxiety and depression-related behaviors [36]. Data from the open-field test and elevated plus maze test in our study also show that *L*. *plantarum* WLPL04 alleviated anxiety and depressive-like behaviors induced by chronic restraint stress, suggesting its antianxiety and antidepression effect of *L*. *plantarum* WLPL04.

The link between microbiome composition and neurodevelopment has been proposed for a long time [4]. Microbiota has been reported to influence the neurodevelopment. The alterations of the gut microbiota may affect the neurodevelopment and could be mediated by microbiota via microbiota-gut-brain axis [37, 38]. Neurodevelopment-related molecular, such as BDNF, has been found to be related with the microbiota-gut-brain axis, and they are susceptible to modulations [4, 39]. The microbiota-gut-brain axis is reported to regulate neuropsychiatric diseases [40]. Alterations of gut microbiota could influence strongly on the neurodevelopment. The *L*. *helveticus* NS8 treatment improves cognitive deficit and anxiety-like behaviors in hyperammonemia rats. The two-month administration of *L*. *plantarum* MTCC1325 ameliorates the cognitive deficits in Alzheimer's disease [16]. The long-term treatment of *Lactobacillus paracasei* K71 may alleviate the age-dependent cognitive decline in mice [41]. The dysbiosis and behavioral deficits caused by prenatal stress can be prevented by treating the dam and the offspring mice with *Lactobacillus*-containing probiotics and indigenous *Lactobacillus reuteri*, respectively [42, 43]. In the present study, the *L. plantarum* WLPL04 treatment rescues cognitive deficits in chronically stressed mice, providing evidence that this probiotic treatment can benefit the host by alleviating stress-induced cognitive disorders.

The stress exposure is known to significantly change the gastric acid secretion, gastrointestinal motility, and mucous levels, which can influence the ability of microbes to colonize within the gastrointestinal tract. The stress can alter the composition of intestinal microbiota. For instance, Bailey et al. have reported that a social stressor reduces the relative abundance of Bacteroidetes in mice [5]. The change in the microbiota diversity can be detected as early as 2 h after stress exposure [44]. A study in nonhuman primates indicates that stress during pregnancy affects the infant gut microbiota by reducing *Bifidobacteria* and *Lactobacilli* [45]. The present study shows a consistent result, indicating that the chronic restraint stress induces changes in bacterial species and diversity.

Although research shows the validity of probiotics, many scholars remain cautious. Suez et al. have found that the potential postantibiotic probiotic benefits may be offset by a compromised gut mucosal recovery [46]. Zmora has confirmed that the empiric probiotic supplementation may be limited and persistently affect the gut mucosa, meriting the development of new personalized probiotic approaches [47]. The effects of probiotic may be dependent on the strain. A probiotic formulation exhibits beneficial effects for stressed animals but has no benefit or may cause harm to normal ones [48]. Lactic acid bacteria are often considered to promote health but are reported to be associated with bad outcomes, including susceptible to inescapable electric stress [49], increased severity of psychotic dysfunctions [50], and high levels of proinflammatory [51]. Lactic acid bacteria are also reported to be relatively abundant in persons with schizophrenia and bipolar disorders [52, 53]. The discrepancy may be explained by differences in study design and methodologies. In the present study, the *L*. *plantarum* WLPL04 is supplied as drinking supplement for chronically stressed mice, and results provide further evidence that this probiotic treatment can rescue the intestinal microbiota composition and support the hypothesis that the *L*. *plantarum* WLPL04 can benefit the host by alleviating stress-induced mood disorders. The 5-HT is produced in the brain and the gastrointestinal tract and can be detected in the blood. Gershon and Tack have reported that the gastrointestinal tract contains most of the body's 5-HT [54]. The synthesis and the metabolism of 5-HT in the brain and periphery are believed to be independent. To our knowledge, 90% of the 5-HT is produced by enterochromaffin cell and stored in granule cells [55]. When stimulus factors are involved, the 5-HT stored in granule cells is released into the blood [56]. The 5-HT produced in the brain cannot pass the blood-brain barrier and cannot enter blood.

The microbiota is involved in regulating the host's 5-HT level. Germ-free mice have significantly low serum serotonin [57]. Approximately 50% of the gut-derived 5-HT is regulated by the gut microbiota. Spore-forming bacteria, which are dominated by the Clostridiaceae and the Turicibacteraceae [57], produce short-chained fatty acids and tryptamine, which influence TPH-1 expression, 5-HT synthesis, and/or 5-HT release [58, 59]. The long-term diet supplementation with *L. paracasei* K71 elevates the serum serotonin level [41]. Consistent with those of the previous studies, our results show that the *L. plantarum* WLPL04 can reverse the serum 5-HT level of chronically restrained mice. The *L. plantarum* WLPL04 may affect the 5-HT synthesis in the gut and rescue the body's 5-HT concentration to a physiological level in the stressed mice.

The serotoninergic system plays an important role in the mood regulation [60]. The 5-HT has long been recognized as a key contributor to the regulation of mood and anxiety and is strongly associated with the etiology of major depression [61]. Early studies demonstrate that disabling the serotonergic system completely either by preventing the 5-HT production or by lesioning the 5-HT neurons leads to widespread behavioral consequences ranging from cognitive deficits to avoidance behaviors [52–65]. Increasing the bioavailability of the 5-HT in serotoninergic synapses effectively alleviates depressive symptoms [66]. In the present study, the serum 5-HT concentration is significantly decreased in the chronically stressed mice, and such decrease is alleviated by the *L. plantarum* WLPL04 treatment.

As the most abundant neurotrophic factor, the BDNF affects several aspects of brain functions. The expression of BDNF is dependent on the activity and regulated by internal and environmental factors. Increasing studies have shown that the stress downregulates the expression of BDNF [67–69]. The intestinal microbiota regulates the level of BDNF in the central nervous system [70]. For example, the administration of antimicrobials transiently alters the composition of microbiota and reduces the expression of BDNF in the hippocampus of mice [69]. Moreover, the hippocampal BDNF expression is upregulated after the mice are orally administered with the probiotic *B*. *longum* 1714 [31]. Consistently, the present study has found that the chronic restraint stress reduces the expression of BDNF and its receptor TrkB in the mPFC and impairs mPFC-dependent cognitive functions, and such effects are alleviated by the supplementation of the *L. plantarum* WLPL04.

## 5. Conclusions

In summary, the *L*. *plantarum* WLPL04 treatment can alleviate anxiety/depressive-like behaviors, the abnormal change in intestinal microbiota, and the reduced levels of 5-HT, BDNF, and TrkB induced by chronic stress, providing an experimental basis for its therapeutic application on anxiety/depressive mood disorders.

## Data Availability

The data used to support the findings of this study are available from the corresponding author upon request.
